# The hunt for efficient, incomplete designs for stepped wedge trials with continuous recruitment and continuous outcome measures

**DOI:** 10.1186/s12874-020-01155-z

**Published:** 2020-11-17

**Authors:** Richard Hooper, Jessica Kasza, Andrew Forbes

**Affiliations:** 1 Queen Mary University of London, London, UK; 2 Institute of Population Health Sciences, Yvonne Carter Building, 58 Turner Street, Whitechapel, London, E1 2AB UK; 3 Monash University, Melbourne, Australia

**Keywords:** Algorithms, Cluster randomised trials, Continuous recruitment, Efficient design, Stepped wedge trials

## Abstract

**Background:**

We consider the design of stepped wedge trials with continuous recruitment and continuous outcome measures. Suppose we recruit from a fixed number of clusters where eligible participants present continuously, and suppose we have fine control over when each cluster crosses to the intervention. Suppose also that we want to minimise the number of participants, leading us to consider “incomplete” designs (i.e. without full recruitment). How can we schedule recruitment and cross-over at different clusters to recruit efficiently while achieving good precision?

**Methods:**

The large number of possible designs can make exhaustive searches impractical. Instead we consider an algorithm using iterative improvements to hunt for an efficient design. At each iteration (starting from a complete design) a single participant – the one with the smallest impact on precision – is removed, and small changes preserving total sample size are made until no further improvement in precision can be found.

**Results:**

Striking patterns emerge. Solutions typically focus recruitment and cross-over on the leading diagonal of the cluster-by-time diagram, but in some scenarios clusters form distinct phases resembling before-and-after designs.

**Conclusions:**

There is much to be learned about optimal design for incomplete stepped wedge trials. Algorithmic searches could offer a practical approach to trial design in complex settings generally.

**Supplementary Information:**

The online version contains supplementary material available at 10.1186/s12874-020-01155-z.

## Background

Stepped wedge trials are cluster-randomised trials where clusters cross over from the control to the active intervention condition during the trial, according to a randomised schedule [1]. Typically, every cluster will begin the trial in the control condition and end in the intervention condition, but the stepped wedge design in the broadest sense allows for more variation than this, including the possibility that some clusters remain in the same condition throughout [2].

The classic schematic representation of a stepped wedge trial shows time separated into regular or discrete “periods” [2], though the reality is that many stepped wedge trials recruit participants continuously over the duration of the trial [3]. Few authors have considered possibilities for the design of stepped wedge trials where cross-over can be scheduled on a continuous time-scale [4]. Another common assumption is that recruitment to a stepped wedge trial occurs in every period in every cluster. But if each new participant increases our research costs, or if we want to minimise the participation burden for ethical reasons (generally true whenever individual consent is required) we may want to consider an “incomplete” design where recruitment effort is focused at particular periods in particular clusters [5].

Suppose we have a fixed number of clusters which recruit participants continuously at a fixed rate over a fixed duration: it may not be necessary to recruit every available participant in order to detect a clinically important effect with given statistical power. Here we are interested in when we should recruit and when we should cross over in different clusters in order to optimise efficiency. We are assuming investigators have a fine degree of control over the timing of recruitment and cross-over at different clusters. One example would be an intervention consisting of a change to a General Practitioner’s computer system, such as an improvement to a dashboard highlighting treatment options for particular patients. Such an intervention could be scheduled seamlessly and electronically.

Although we are interested in situations where eligible participants present as a random process in continuous time, we will simplify by imagining that eligible participants arrive at each of *K* clusters at regularly-spaced times 1/*M*, 2/*M*, …, *M*/*M* (where *M* is the rate of arrival at each cluster and the total duration of recruitment is scaled to be one time unit). We assume *K* and *M* are fixed or constrained by design – in other words that we have a certain number of clusters available to us, and a certain length of time. Our interest in this article is particularly on designs with large *K* (triallists are often cautioned against running cluster-randomised trials with small numbers of clusters) [6], and large *M* (so that we have a reasonable model for a continuous time process).

We suppose that we can choose whether or not to recruit each eligible participant. Assuming a linear model for outcomes we can then determine, for any given design, the precision of the treatment effect estimate using standard results [7–9], and compare different designs. As *M* increases, however, the number of possible designs becomes very large, and calculating the precision of each design requires us to invert an increasingly large matrix using numerical methods. Collapsing the data to the cluster-period level (a trick often employed with longitudinal cluster randomised trials in discrete time) is not possible here because neither the time effect nor the intra-cluster correlation in our model is piecewise-constant within “periods”. As such, the vector of cluster-period means is not a sufficient statistic for the treatment effect [10].

In this article we present a computational algorithm to help researchers select an efficient design. Though the algorithm is not guaranteed to find an optimal solution it is driven by rules aimed at maximising the precision with which the treatment effect is estimated. It is also replicable and much faster than enumerating all possible designs. Our aim was to improve understanding of the form of optimal designs for incomplete stepped wedge trials in continuous time, and to demonstrate proof of principle for the use of computational methods in solving complex design problems.

## Methods

### Statistical model

Suppose that participant *i* = 1, …, *m*_*k*_ in cluster *k* = 1, …, *K* is recruited at time *t*_*ik*_. We assume that each participant has the primary outcome assessed once, at a fixed time following recruitment. The outcome for participant *i* in cluster *k* is *Y*_*ik*_, which we assume to be on a continuous scale of measurement. A “design” consists of a schedule for recruitment at each cluster (which determines the *t*_*ik*_ and the numbers of participants recruited at different clusters, *m*_*k*_), and a schedule for cross-over. For simplicity, we will assume that recruitment under the intervention condition can follow seamlessly from recruitment under the control condition, i.e. that there is no need for a transition period to allow for implementation of the intervention condition [1, 3], and that a cluster can cross over to the intervention from time 0 onwards. One advantage of the numerical and computational approaches used in this article is that they could readily be adapted to a different design problem where a transition period (during which recruitment is suspended) must be included between periods of recruitment under the control and recruitment under the intervention. Suppose cluster *k* crosses over from the control to the active intervention condition at time ${t}_k^{\ast }$.

Our model for the outcome is:

$$ {Y}_{ik}=T\left({t}_{ik}\right)+\theta H\left({t}_{ik}-{t}_k^{\ast}\right)+{\varepsilon}_{ik}, $$

where *H*(*t*) is a step function taking the value 1 if *t* ≥ 0 and 0 otherwise, and the parameter *θ* is the treatment effect we would like to estimate. This implies that the treatment has an immediate effect when introduced at a cluster – that is, an effect on all new participants who subsequently present at that cluster. This is plausible if the implementation is itself instantaneous, with no need for a transition period, as we have assumed here [3].

*T*(*t*) is a function representing the fixed effect of time on outcome (common to all clusters). This fixed time effect could include secular, periodic, or even discontinuous changes (though it seems artificial and implausible to assume that the time effect is piecewise constant, even if there are meaningful, a priori divisions of the time-scale at which to place the discontinuities). In this article we will assume a polynomial time effect.

The important thing for our present purposes is to model the time effect appropriately, whatever form that relationship takes. We are not so interested here in how to make a robust analysis choice when the form of the relationship is unknown, which could involve alternatives such as restricted cubic splines. Polynomial time effects were particularly simple and fast to encode as a design matrix when running our code for calculating the variance of the treatment effect estimator, which was appealing for practical reasons since the solutions we sought required iterating this code a great many times. They also offered a simple way to explore what happened when the time effect was made less smooth (using a polynomial of higher degree). How well an analysis would perform if the time effect were misspecified is certainly of some interest, but this would require simulation and we leave this to future work: here we focus on design rather than analysis.

The *ε*_*ik*_ in the model are random errors, each with mean 0 and variance *σ*^2^. Clustering implies that the errors *ε*_*ik*_ are correlated within clusters: we will allow for the possibility that these correlations decay smoothly as the separation in time increases [10, 11]. Specifically, we consider the following correlation structure:

$$ {\displaystyle \begin{array}{c}\mathrm{Corr}\left({\varepsilon}_{i_1k},{\varepsilon}_{i_2k}\right)={\rho \tau}^{\left|{t}_{i_1k}-{t}_{i_2k}\right|},\\ {}\mathrm{Corr}\left({\varepsilon}_{i_1k},{\varepsilon}_{i_2l}\right)=0,\kern0.5em \mathrm{if}\ k\ne l,\end{array}} $$

$$ \mathrm{Var}\left({\varepsilon}_{ik}\right)={\sigma}^2. $$

Here *ρ* represents the intracluster correlation for two individuals sampled at the same time from the same cluster, and *τ* represents the decay in this correlation with increasing separation in time. Time runs from 0 to 1, hence the correlation between the outcomes of two different individuals from the same cluster sampled at either end of the entire trial period is *τρ*. Different individuals sampled from the same cluster have been observed in real-life cohorts to have less strongly correlated outcomes the further apart in time from each other that they are sampled [4, 10, 12].

Our model generalises the discrete-time model of Hussey and Hughes to continuous time [7].

The precision of the treatment effect estimator is calculated using standard results, assuming a generalised least squares approach to estimation [7–9]. Formally, if we write outcomes *Y*_*ik*_ as a single column vector **Y**, and parameters for fixed effects (including time effects and treatment effect) as a column vector **θ**, and express the linear model above in matrix form

$$ \mathbf{Y}=\mathbf{Z}\boldsymbol{\uptheta } +\mathbf{e},\kern0.75em \mathbf{e}\sim \mathrm{N}\left(\mathbf{0},\mathbf{V}\right), $$

then the variance of the generalised least squares estimator for **θ** is

$$ \mathrm{Var}\left(\hat{\boldsymbol{\theta}}\right)={\left({\mathbf{Z}}^{\prime }{\mathbf{V}}^{-1}\mathbf{Z}\right)}^{-1}. $$

### Symmetry

Observe that the correlation structure described above is unchanged if we run time backwards. Now, it is not the case that if we reverse the time-scale of a stepped wedge trial design we end up with another valid stepped wedge design (because in the time-reversed design some clusters would be asked to cross from the intervention back to the control). But suppose we take a given stepped wedge design, reverse the time-scale (that is, transform *t* to 1 − *t*), and also swap the control and the intervention conditions everywhere. Then the result *is* a valid stepped wedge design – and both the original design and the design with time and condition reversed will estimate the treatment effect with exactly the same precision.

Motivated by this symmetry, and in order to simplify the space of designs over which we conduct our search, we restrict attention to designs that are invariant under a time-and-condition-reversing transformation. The “classic” stepped wedge design in which clusters all start in the control condition, finish in the intervention condition, and cross over at regularly-spaced intervals, is an example of this kind of invariance.

To achieve this symmetry we assume an even number of clusters, *K*, and work on the schedules for recruitment and cross-over in half of the clusters only, *k* = 1, 2, …, *K*/2. The timing of recruitment and cross-over in the remaining half of the clusters, starting from the last cluster and counting back, *K*, *K* − 1, …*K*/2 + 1, is then set to match the first half but with time reversed.

### The algorithm

As an approximation for a continuous time process we assume that a new eligible participant arrives at each of *K* clusters at regularly-spaced times 1/*M*, 2/*M*, …, *M*/*M*.

For a given sample size (the total number of participants recruited under a given design) the algorithm looks for small, incremental design changes that improve precision while preserving sample size – for example by shifting a cross-over time or rearranging the recruitment schedule (the changes considered are described in more detail in the supplementary Appendix). These modifications continue until no additional improvements can be found. To reduce (or increase) the sample size the algorithm then removes (or adds) the one participant who is calculated to make the least (or most) difference to the precision. (This is a development of previous work in the discrete-time context, proposing that the information content of each cluster-period in a complete design could be quantified as the loss of precision when recruitment is suspended in that cluster period.) [13, 14]

These steps are integrated into the overall algorithm in the following way. Step 1: start with a complete design with sample size *MK*. Step 2: modify the design to improve the precision without altering sample size (shifting cross-over times or rearranging the recruitment schedule). Step 3: remove the participant calculated (with an exhaustive search) to make the least difference to precision. Step 4: modify the design to improve the precision without altering sample size. Step 5: return to Step 3.

In this way a solution can be obtained for every possible sample size. If our target is a design which detects a minimal clinically important treatment effect *δ* with given statistical power or precision, then we need only run the algorithm until we reach a sample size that just fails to achieve this precision, and then adopt the design from the previous iteration of the algorithm.

To guard (to some extent) against the possibility that the algorithm is drawn to a local rather than global optimum in the space of possible designs, we then repeat the algorithm, but from a different starting point and now working in the opposite direction. Step 1: start with a simple design with small sample size (see below). Step 2: modify the design to improve precision without altering sample size. Step 3: *add* the participant calculated to make the most difference to precision. Step 4: modify the design to improve precision without altering sample size. Step 5: return to Step 3. This continues until we reach a sample size and design that achieve the required precision. If the solutions obtained by working from our two starting points differ, the algorithm selects the design that achieves the target precision with smaller sample size.

Our starting points for the forward and backward searches are illustrated in the supplementary Appendix. The starting point for the forward search is a complete design where the cross-over boundary follows a straight, diagonal line – that is, in cluster *k* the last time-point in the control condition (before cross-over to the intervention) is *i*/*M*, where *i* is the nearest integer to *M*(*k* − 1)/(*K* − 1). The starting point for the backward search is a “staircase” design [13], which we define here as a design with the same cross-over boundary as the starting point for the forward search, but with recruitment only at a fixed number of time-points *j* immediately before cross-over and the same number immediately after cross-over. For the design which begins our forward search we set *j* to be the nearest integer to *M*/(*K* − 1).

### Software and code

We coded the algorithm in Mata, within Stata v16 (StataCorp, College Station, TX USA), which is a compiled language offering rapid execution. The matrix inversion needed to calculate precision for each design uses Mata’s built-in ‘invsym’ function, but also takes advantage of the fact that the inverse of a block-diagonal matrix (such as the correlation matrix between all pairs of outcomes *Y*_*ik*_) is itself a block-diagonal matrix [7]. Code is available from a GitHub repository (https://github.com/richard-hooper/incomplete-continuous-SWT).

### Scenarios investigated

We consider the illustrative case of a trial with 30 clusters and 100 eligible participants in each cluster, and with the following correlation structures: (i) *ρ* = 0.01, *τ* = 1.0; (ii) *ρ* = 0.05, *τ* = 0.2; (iii) *ρ* = 0.05, *τ* = 1.0; (iv) *ρ* = 0.25, *τ* = 0.2; (v) *ρ* = 0.25, *τ* = 1.0. We assume in the first instance that the time effect is a sixth degree polynomial, but we also discuss the impact of the functional form of the time effect on the choice of design.

Our target in each case is to achieve a design which minimises the sample size needed to detect a given effect size with 90% power at the two-sided 5% significance level. We consider effect sizes (*δ*/*σ*) of 0.15, 0.20, 0.25, 0.30, and 0.35. In those instances where even a complete design is not powerful enough to detect the given effect size, we illustrate the most powerful complete design that the algorithm can find.

### Comparison with randomly generated and staircase designs

To provide some context we compare the performance of the algorithm’s solutions for different sample sizes with the performance of (i) 100,000 randomly generated designs, and (ii) staircase designs (as defined above) with different widths of the recruitment window before and after cross-over, in the illustrative scenario *K* = 30, *M* = 100, *ρ* = 0.05, *τ* = 0.2. Randomly generated designs were chosen to be invariant under a time-and-condition-reversing transformation (achieved by randomly generating treatment and recruitment schedules for half the clusters, and then reversing these schedules in the other half). Random cross-over times in different clusters were independent and uniformly distributed, and each participant was assumed to be recruited independently of any other with probability *p*, where *p* was fixed for a given design and distributed uniformly over [0,1] between designs.

## Results

**Fig. 1:**
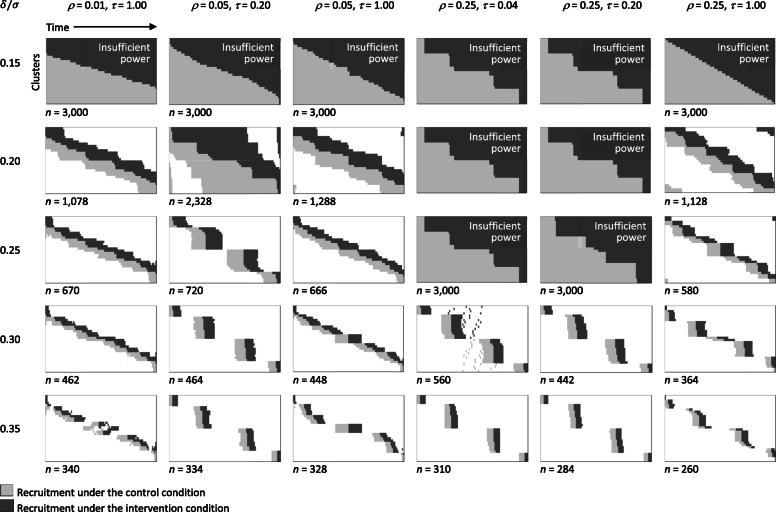
Trial designs that achieve 90% power at the two-sided 5% significance level to detect various effect sizes, *δ*/*σ*, for different combinations of *ρ* (the intracluster correlation for two individuals sampled at the same time from the same cluster) and *τ* (representing the decay in this correlation over the duration of the trial). Designs assume *K* = 30 clusters which are arranged down the vertical axis of each schematic, and *M* = 100 eligible participants presenting in each cluster, arranged along the horizontal axis. Underneath each schematic is the overall sample size. In those instances where even a complete design is not powerful enough to detect the given effect size, we illustrate the most powerful complete design that the algorithm can find

The designs obtained by the algorithm under each scenario are shown in Fig. 1 (the patterns are also available in spreadsheet form from the GitHub repository cited above).

The complete solutions in cases where *τ* = 1.0 resemble the “hybrid” designs described by Girling & Hemming [15], in which the cross-over boundary between recruitment under the control and intervention conditions is roughly linear. The complete solution for *ρ* = 0.05, *τ* = 0.2 has a slightly more serpentine boundary between control and intervention, while the complete solutions for *ρ* = 0.25, *τ* = 0.2 and *ρ* = 0.25, *τ* = 0.04 arrange clusters in four macro-steps. The latter two scenarios are also notable for being less well powered than others: even a complete design is underpowered in these instances for detecting an effect size of 0.25.

As we increase the effect size we want to detect, the required sample size decreases. Incomplete design solutions in Fig. 1 generally focus recruitment around the cross-over boundary, though there are notable instances where this is complemented by recruitment in the off-diagonal corners of the diagram. It is also noteworthy that patterns of recruitment in some scenarios exhibit high-level structure. This is particularly striking in the designs to detect effect sizes of 0.30 and 0.35 for the cases where *τ* < 1.0. Here the clusters are arranged in four groups, in each of which the comparison between control and intervention appears to be almost entirely within (rather than between) clusters.

**Fig. 2:**
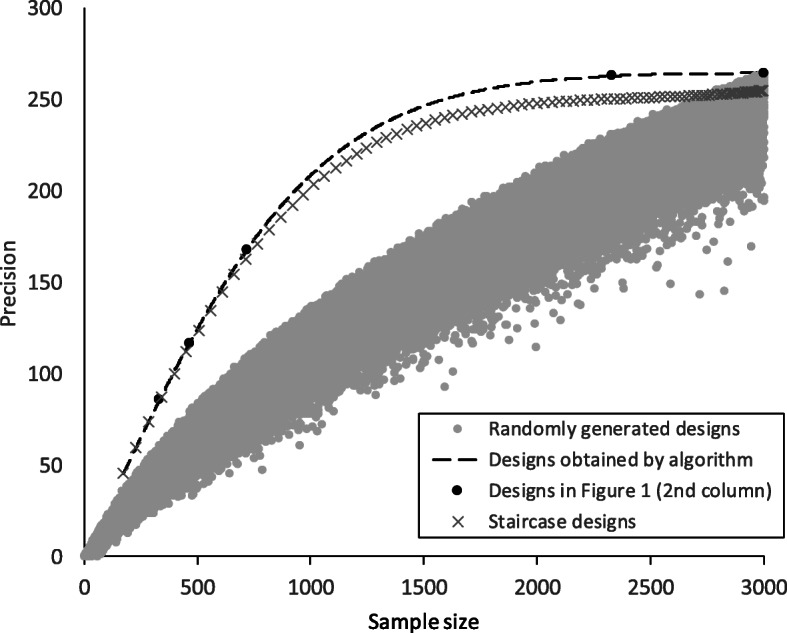
Precision (inverse of the variance of the treatment effect estimator, assuming that the variance of observed outcomes, *σ*^2^, is 1) against sample size for different designs, in the case of *K* = 30, *M* = 100, *ρ* = 0.05, *τ* = 0.2. The dashed line shows designs obtained by the algorithm for different sample sizes (with dark-filled circles indicating the designs shown in the second column of Fig. 1). Pale-filled circles denote 100,000 randomly generated designs. Crosses show the performance of staircase designs with varying width of recruitment window

Figure 2 plots the performance of the algorithm’s solutions in comparison with randomly generated and staircase designs, in the illustrative scenario *K* = 30, *M* = 100, *ρ* = 0.05, *τ* = 0.2 (represented in the second column of Fig. 1). The plot shows the precision of the treatment effect estimator, where precision is defined as the inverse of the variance of the estimator, assuming that the variance of observed outcomes, *σ*^2^, is 1. In this example the relative precision of a staircase design compared with a design with the same sample size obtained by the algorithm is never less than 95%. The dashed line showing the precision of the algorithm’s solutions illustrates the trade-off that can be achieved between sample size and precision: for small sample sizes the precision is roughly proportional to the sample size, but then begins to level off as the sample size increases. By the time the sample size has reached just 50% of the maximum available the precision is already 93% of what is achievable with a complete design.

### The functional form of the time effect

**Fig. 3:**
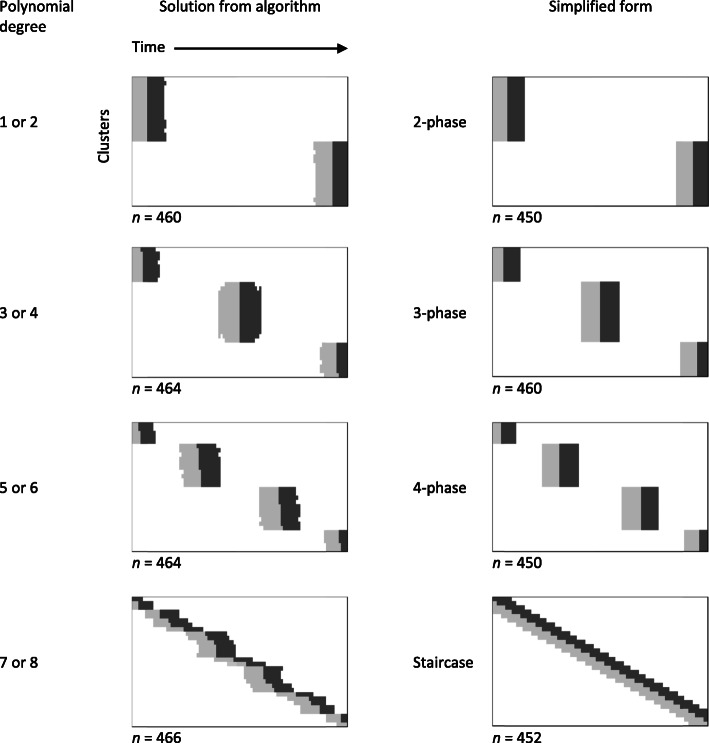
Solutions obtained from the algorithm when the time effect is modelled as a polynomial of degree 1 up to 8, in the case of *K* = 30, *M* = 100, *ρ* = 0.05, *τ* = 0.2, *δ*/*σ* = 0.3. Simplified design forms are also presented

For the results presented above we assumed a sixth degree polynomial for the time effect. This might seem an excessive number of degrees of freedom to use up in practice in estimating the time effect, though not in comparison with stepped wedge trials where the time effect is modelled as a categorical (i.e. discrete) variable with a different level for each design period. To investigate the influence of the functional form of the time effect, we also considered polynomials of degree 1 up to 8 in the illustrative scenario *ρ* = 0.05, *τ* = 0.2, and *δ*/*σ* = 0.3. The designs obtained by our algorithm in each case are shown in Fig. 3. With both linear and quadratic effects of time, the algorithm settles on the same, two-phase before-and-after design. Cubic and quartic effects of time both lead to the same, three-phase design. Polynomial degree 5 leads to the same, four-phase design as degree 6, while degrees 7 and 8 produce a design where the phases are less distinct – closer in appearance to a staircase design.

In practice, of course, we may have little to guide us a priori in choosing an appropriate functional form for the time effect. What if we went ahead with one of the designs in the first column of Fig. 3 (or, more realistically, some simplified design form with similar total sample size, as illustrated in the second column of Fig. 3), but concluded a posteriori that we needed to adjust for a different functional form? We evaluated the power to detect an effect *δ*/*σ* = 0.3 (at the 5% significance level) of each simplified design in Fig. 3 under different polynomial forms for the time effect, with *ρ* = 0.05, *τ* = 0.2. The results are shown in Table 1. The power of the multi-phase designs depends heavily on accurate selection of the degree of the polynomial, and consequently the number of phases: if there are fewer phases than are appropriate to the degree of the polynomial then the power drops dramatically. The staircase design performed well over the whole range of polynomial degrees.

**Table 1:** Statistical power (at the two-sided 5% significance level) of the simplified design forms shown in Fig. 3, under different models for the time effect (polynomials of degree 2, 4, 6 or 8), to detect effect size *δ*/*σ* = 0.3, with *K* = 30, *M* = 100, *ρ* = 0.05, *τ* = 0.2

	Polynomial degree			
Simplified form	2	4	6	8
2-phase	89%	37%	35%	22%
3-phase	90%	90%	37%	33%
4-phase	89%	89%	89%	43%
Staircase	89%	89%	89%	89%

## Discussion

Some of our findings were expected. Hybrid designs have been shown to be asymptotically optimal among complete designs in the discrete time case, at least when *τ* = 1.0 (i.e. when there is no decay in the intracluster correlation over time) [15]. Our results suggest something similar when time is treated as a continuous phenomenon. For an optimal incomplete design it is not surprising that recruitment in a cluster should be concentrated just before and just after cross-over: any decay in the intracluster correlation will mean that within-cluster comparisons are made most powerfully over a short timescale like this. What is more surprising is that we should see designs in Fig. 1 that include recruitment in the off-diagonal corners of the diagram in addition to the leading diagonal, though a similar pattern has been noted before in the context of discrete-time models [13].

The other surprising feature of some of the designs in Fig. 1 is the separation into distinct phases of recruitment, with little overlap of control and intervention recruitment periods in different clusters, as if clusters were being randomised to phases of a multi-phase before-and-after study. This pattern is observed for larger effect sizes when *τ* < 1.0. The concentration of recruitment at certain times – and in particular the increase in the number of these distinct phases of data collection with increasing degree of polynomial for the time effect – is reminiscent of results from the literature on the optimal design of experiments for polynomial regression on a continuous variable [16]. This should not surprise us. Although it is the treatment effect that interests us most directly, a design that relies heavily on before-and-after comparisons to estimate this treatment effect must also model the underlying time effect as precisely as possible.

Note that a wholly before-and-after design was always going to be an admissible solution once we focused on time effects with a form that was not confounded with treatment. In our case a smoothly varying (polynomial) effect of time is distinguishable from a discontinuous jump in outcomes when the intervention is introduced, even in a wholly before-and-after design.

We chose particular values of *K* and *M* to illustrate the design problem and its solutions. The approach presented in this article and the supplementary material could be used to investigate other scenarios. Further analytical work is needed to clarify asymptotic relationships of *K* and *M* with the form of the patterns seen in Fig. 1, and thus to draw more generalisable conclusions.

We cannot rule out the possibility that a solution obtained by the algorithm is a local optimum in the design space rather than a global optimum, although our intuition about what optimal designs look like may be equally unreliable. Figure 2 illustrates nicely just how well the algorithm performs in identifying efficient designs, with performance well beyond the envelope generated by randomly selected designs.

None of the designs in Fig. 1 is an exact staircase design in the sense we have used the term, but Fig. 2 shows that staircase designs perform well over the gamut of sample sizes in the scenario illustrated. They may also cope well with a variety of functional forms for the time effect. The focus of the present article is on an algorithmic search for an efficient design (whatever that looks like), but staircase designs will undoubtedly repay further investigation in the study of incomplete stepped wedge trial designs.

We simplified considerably in assuming that eligible participants present at regular, fixed intervals rather than as a random continuous-time process, but assuming that the arrival rate is constant over time we would expect arrival times in a sample to become increasingly uniformly spread as *M* increases. Simulation studies investigating the impact of unevenly spaced arrival times on the precision of complete stepped wedge designs suggest that this impact is small [11].

Finally, note that we have been working within a generalised least squares estimation framework, and are envisaging analysis using mixed regression or generalised estimating equations, as often recommended as a starting point for the analysis of stepped wedge trials [1]. With different approaches, for example robust estimation methods that reduce reliance on modelling assumptions [17, 18], it may be that different patterns emerge.

## Conclusions

This article is not an invitation to conduct before-and-after studies in place of randomised trials. Nevertheless, the idea that a kind of multi-phase, interrupted time series study might perform well in certain scenarios is intriguing and appealing. Note that multi-phase before-and-after designs, such as those illustrated in Fig. 3, still feature randomisation in that clusters are randomised to phases. These designs depend heavily on modelling to allow for time effects, but offer a simple and straightforward plan for conducting an evaluation. The less smooth you think the time effect might be, the more phases you should schedule. Staircase designs may offer a more robust approach to incomplete trial design, but will require more complex scheduling.

We have illustrated how a computational approach could help with the search for an efficient trial design when this problem is otherwise intractable, though we should be wary of the possibility that an algorithm such as ours, which proceeds by small, incremental changes, might become attracted to a local optimum in the design space rather than a global one. Other approaches, including stochastic searches [19], may be worth investigating. Finally, we recognise that trial design in the real world must consider practicality, simplicity and constraints on resources as much as numerical efficiency.

## Supplementary Information

**Additional file 1.**

## Data Availability

Supplementary materials, including code, are available from a GitHub repository at https://github.com/richard-hooper/incomplete-continuous-SWT.
