# Supplementary material for: The hunt for efficient, incomplete designs for stepped wedge trials with continuous recruitment and continuous outcome measures
Source: BMC Med Res Methodol. 2020 Nov 17;20:279. doi: 10.1186/s12874-020-01155-z (PMC7672921; doi:10.1186/s12874-020-01155-z)
Supplement: Supplementary file 1 — Additional file 1. [file 12874_2020_1155_MOESM1_ESM.pdf]

Optimal incomplete designs for stepped wedge trials in continuous time

Richard Hooper[1], Jessica Kasza[2], Andrew Forbes[2]

[1]Queen Mary University of London, London, UK
[2]Monash University, Melbourne, Australia

APPENDIX: How the algorithm works

The starting point for the backward search algorithm is a complete design with classic stepped wedge form, where the cross-over boundary follows a straight line from the top-left corner to bottom-right, and the first and last clusters remaining in the same treatment condition (control and intervention, respectively) for the full duration of the trial. The starting point for the forward search algorithm is a staircase design having the same cross-over boundary as the classic stepped wedge, but with recruitment concentrated just before and just after cross-over, for just long enough to overlap with the previous and the next sequences. These starting points are illustrated below for the case $K = 30, M = 100$:

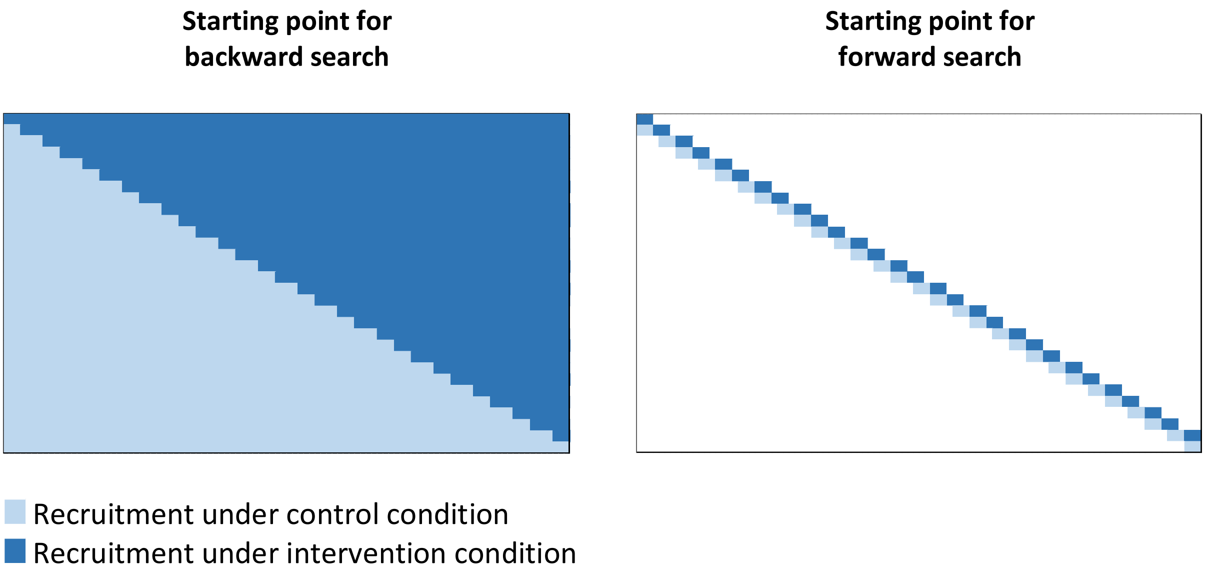

We restrict attention to designs that are invariant if time is reversed and the control and intervention conditions are swapped. To achieve this the algorithm manipulates only the first half of the clusters in the design. The second half is then obtained from the first half by reversing time and swapping control and intervention.

The main steps in the algorithm are summarised in the flow-charts over the page. If the forward and backward searches arrive at different designs then the algorithm selects the solution with the greater precision.

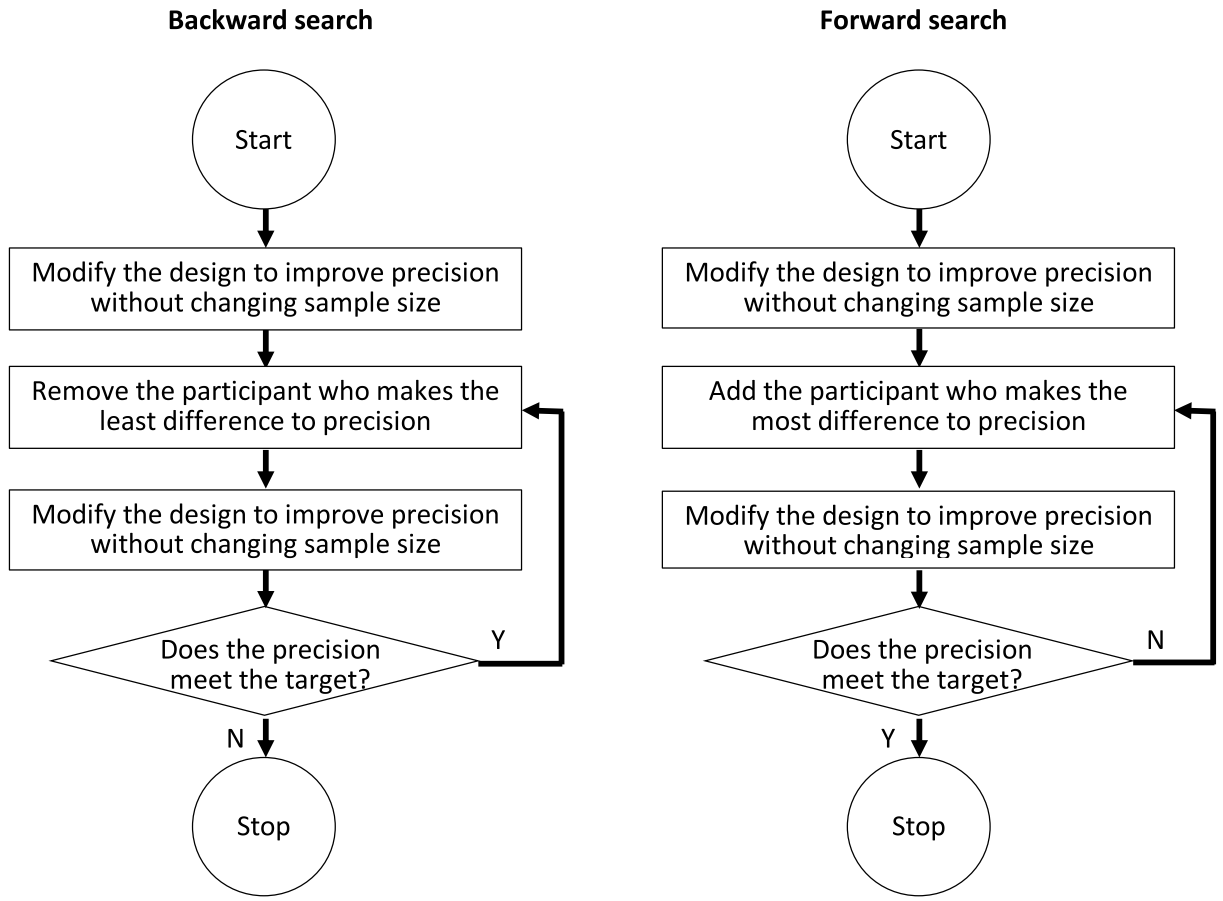

Possible modifications for improving precision without changing sample size are described in the box below. The algorithm considers each cluster, working from top to bottom, and chooses the optimal modification to the sequence for each cluster from the options given in the box below, and then repeats this cycle until no further improvements are possible.

Sample size-preserving modifications of a given sequence ($M = 50$):

1. Shifting cross-over left or right by one individual,* but with recruitment schedule unchanged:

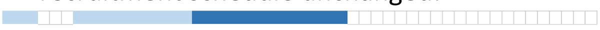

becomes

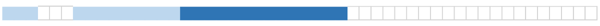

or

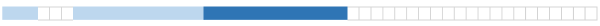

2. Shifting recruitment schedule left or right by one individual, but with cross-over unchanged:

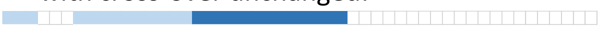

becomes

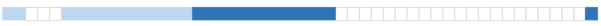

or

In instances like this one the recruitment schedule wraps around at either end of the time-line to preserve total sample size. This is intentional: previous work by Kasza & colleagues (*Biometrics* 2019;75:144-152) suggests that having recruitment at both ends of the time-line in some clusters can be advantageous.

3. Shifting recruitment schedule and cross-over left or right by one individual:*

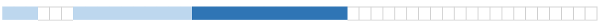

becomes

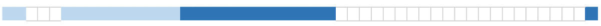

or

* Cross-over shifts are only allowed if the cross-over boundary for the whole design remains monotonic.
